# IGF-1R inhibition induces MEK phosphorylation to promote survival in colon carcinomas

**DOI:** 10.1038/s41392-020-0204-0

**Published:** 2020-08-26

**Authors:** Qing Wang, Yan Zhang, Jiang Zhu, Honggang Zheng, Shuntai Chen, Li Chen, Hsin-Sheng Yang

**Affiliations:** 1 Department of Toxicology and Cancer Biology, College of Medicine, University of Kentucky, Lexington, KY USA; 2 Department of Breast Surgery, Qilu Hospital of Shandong University, Jinan, Shandong China; 3 Department of Oncology, Guang’anmen Hospital, China Academy of Chinese Medical Sciences, Beijing, China; 4 Markey Cancer Center, College of Medicine, University of Kentucky, Lexington, KY USA; 5 Present Address: Department of Breast Surgery, Qilu Hospital of Shandong University, Jinan, Shandong China

**Keywords:** Cancer therapy, Gastrointestinal cancer

## Abstract

The insulin-like growth factor 1 receptor (IGF-1R) governs several signaling pathways for cell proliferation, survival, and anti-apoptosis. Thus, targeting IGF-1R appears as a reasonable rationale for tumor treatment. However, clinical studies showed that inhibition of IGF-1R has very limited efficacy due to the development of resistance to IGF-1R blockade in tumor cells. Here, we discovered that prolonged treatment of colon cancer cells with IGF-1R inhibitors (BMS-754807 and GSK1838705A) stimulates p70 KDa ribosomal protein S6 kinase 1 (p70S6K1) activation, a well-known kinase signaling for cell survival. We also found that p70S6K1 activation by IGF-1R inhibition is independent of K-Ras and PIK3CA mutations that frequently occur in colon cancer. Besides the increased p70S6K1 phosphorylation, the phosphorylation of mitogen-activated protein kinase kinase 1 and 2 (MEK1/2) was elevated in the cells treated with BMS-754807. Interestingly, the increases in MEK1/2 and p70S6K1 phosphorylation were also observed when cells were subjected to the treatment of AKT inhibitor or genetic knockdown of AKT2 but not AKT1, suggesting that AKT2 inhibition stimulates MEK1/2 phosphorylation to activate p70S6K1. Conversely, inhibition of MEK1/2 by MEK1/2 inhibitor (U0126) or knockdown of MEK1 and MEK2 by corresponding *mek1* and *mek2* siRNA enhanced AKT phosphorylation, indicating mutual inhibition between AKT and MEK. Furthermore, the combination of BMS-754807 and U0126 efficiently decreased the cell viability and increased cleaved caspase 3 and apoptosis in vitro and in vivo. Our data suggest that the treatment of colon tumor cells with IGF-1R inhibitors stimulates p70S6K1 activity via MEK1/2 to promote survival, providing a new strategy for colorectal cancer therapeutics.

## Introduction

The insulin-like growth factor 1 receptor (IGF-1R) belongs to the tyrosine kinase receptor family. By binding with IGF-1, IGF-2, and insulin, IGF-1R activates downstream pathways including phosphoinositide 3 kinase (PI3K)/AKT and Ras/extracellular signal-regulated protein kinase (ERK) pathways,^1^ two frequently activated pathways in colorectal cancer. Elevation of IGF-1R expression or activity is frequently found in many types of cancer, which is associated with tumor cell proliferation, survival, anti-apoptosis, and drug resistance.^2^ For example, IGF-1R-mediated activation of AKT, in turn, phosphorylates and inhibits pro-apoptotic proteins such as Bad and caspase 9 to promote survival.^3,4^ In colorectal cancer, IGF-1R expression levels are commonly elevated in cancerous tissues relative to adjacent normal tissues.^5^ Most importantly, the elevation of IGF-1R expression is associated with a worse prognosis in colorectal cancer patients.^6^ Since the IGF-1R signaling pathway is critical for the development, maintenance, and survival of cancers, many small molecule compounds and antibodies targeting IGF-1R have been developed. Unfortunately, these anti-IGF-1R agents have very limited efficacy in clinical trials,^7–9^ suggesting the existence of a mechanism to antagonize the IGF-1R inhibition in cancer cells.

Previously, we found that prolonged treatment of colon cancer cells with an IGF-1R inhibitor, OSI-906, induces p70S6K1 activation.^10^ The induction of p70S6K1 activation in colon cancer cells is associated with resistance of IGF-1R inhibition since knockdown of p70S6K1 or inhibition of p70S6K1 activity enhances the cell sensitivity to IGF-1R inhibition. p70S6K1 is a serine/threonine kinase, which is the key downstream point of the AKT-mTOR pathway.^11^ p70S6K1 activation has been shown to boost cell survival.^11^ The well-known mechanism for p70S6K1 to promote survival is that p70S6K1 phosphorylates mouse double minute 2 (MDM2) leading to polyubiquitination and degradation of p53, and thus represses p53-dependent apoptosis.^12^ Interestingly, p70S6K1 is one of the downstream targets of AKT. Inhibition of AKT activation by IGF-1R inhibitor, OSI-906, is supposed to suppress p70S6K1 activation. However, colon tumor cells treated with OSI-906 suppresses AKT activation but induces p70S6K1 phosphorylation.^10^ This finding raises the question of whether the activation of p70S6K1 by OSI-906 is through IGF-1R inhibition or it is a non-specific effect of OSI-906. If it is through IGF-1R inhibition, what is the molecular mechanism to induce p70S6K1 activation?

We also discovered that the level of the tumor suppressor, Programmed cell death 4 (Pdcd4), correlates with the chemosensitivity to OSI-906 in colon tumor cells.^10^ Colon tumor cells with high Pdcd4 expression is sensitive to OSI-906 inhibition, while Pdcd4 knockdown gains the resistance to OSI-906 inhibition.^10^ Conversely, colon cells with low Pdcd4 level are resistant to OSI-906 inhibition and this resistance is relieved by Pdce4 overexpression.^10^ Pdcd4 has been demonstrated to inhibit tumor promotion and progression,^13^ whose expression is often downregulated in colorectal cancer.^14,15^ Pdcd4 physically interacts with eukaryotic initiation factor 4A (eIF4A) and attenuates protein translation.^16^ eIF4A is an ATP-dependent RNA helicase belonging to DEAD-box RNA helicase protein family.^17^ eIF4A mutants that are defective in RNA helicase activity inhibits translation of mRNAs with stable secondary structures more efficiently than less-structured mRNAs,^18^ indicating that eIF4A’s helicase activity is required for translation of structured mRNAs. Thus, inhibiting eIF4A activity by Pdcd4 is expected to suppress protein translation, especially the translation of structured mRNAs. This notion was recently confirmed by identification of the Pdcd4 target, stress-activated-protein kinase interacting protein 1 (Sin1), whose translation is inhibited by Pdcd4 through suppressing eIF4A activity.^19^

In this study, we intend to resolve the mechanism of induction of p70S6K activation by IGF-1R inhibition using a different IGF-1R inhibitor, BMS-754807. BMS-754807 is a potent IGF-1R inhibitor, which has been reported to have a limited off-target effect since the transcriptional profile is similar between wild-type MEF cells treated with BMS-754807 and IGF-1R null MEF cells.^20,21^ We found that colon tumor cells treated with an IGF-1R inhibitor, BMS-754807 or GSK1838705A, induced phosphorylation of p70S6K1 as OSI-906 does. We also found that Pdcd4 inhibited the induction of p70S6K1 phosphorylation by IGF-1R inhibitor was through suppression of p70S6K1 translation. In addition, BMS-754807 treatment induced phosphorylation of mitogen-activated protein kinase kinase 1 and 2 (MEK1/2), which contributed to BMS-754807-induced p70S6K1 activation for survival in colon cancer cells. These findings provide new mechanistic insights into combating the resistance of IGF-1R inhibition.

## Results

### Induction of p70S6K1 phosphorylation by IGF-1R inhibitor is independent of K-Ras and PIK3CA mutations

**Fig. 1:**
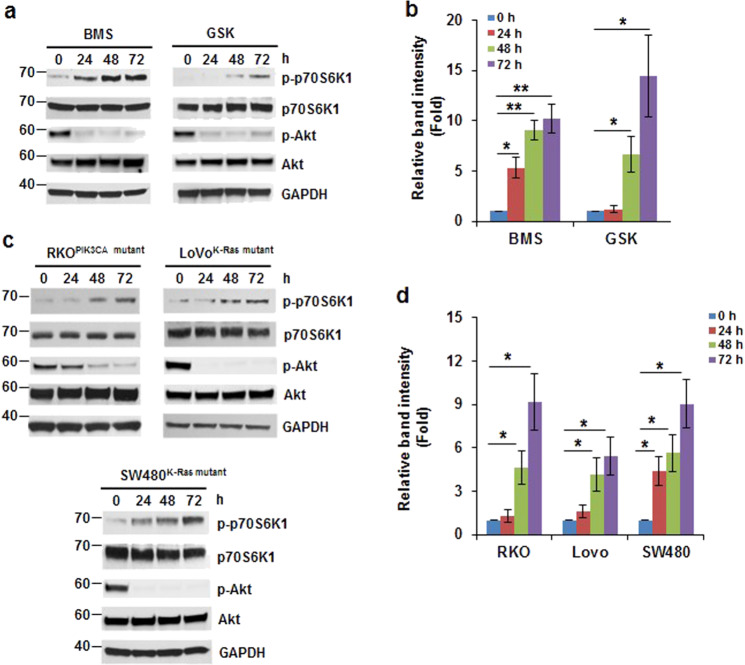
Induction of p70S6K1 phosphorylation by IGF-1R inhibitors. **a**, **b** IGF-1R inhibitors induced p70S6K1 phosphorylation in HCT116 cells. Cells were treated with BMS-754807 (240 nM, BMS) or GSK1838705A (2 μM, GSK) for 0–72 h. **a** Representative western blot images are shown. **b** Densitometric quantification of the western blot results in (**a**). For each inhibitor, the band intensity of p-p70S6K1/GAPDH with 0 h treatment is designated as 1. Data from three independent experiments were analyzed by one-sample *t*-test (mean ± SD; *P < 0.05; **P < 0.01). **c**, **d** The induction of p70S6K1 phosphorylation by IGF-1R inhibition is independent of K-Ras or PIK3CA mutations. Cells were treated with BMS-754807 (240 nM) for 0–72 h. **c** Representative western blot images are shown. **d** Densitometric quantification of western blot results in (**c**). The band intensity of p-p70S6K1/GAPDH with 0 h treatment in each cell line is designated as 1. Data from three independent experiments were analyzed by one-sample *t*-test (mean ± SD; *P < 0.05)

Previously, we demonstrated that treatment of HCT116 and SW480 cells with an IGF-1R inhibitor, OSI-906, increases phosphorylation of p70S6K1.^10^ To test whether the induction of p70S6K1 phosphorylation is due to inhibition of IGF-1R signaling pathway not non-specific outcome of OSI-906, the HCT116 cells were treated with other IGF-1R inhibitors, BMS-754807 and GSK1838705A, for 0–72 h. Since IGF-1R inhibition results in suppression of downstream AKT activation, the IGF-1R inhibitors (BMS-754807 and GSK1838705A) efficiently inhibit AKT phosphorylation, indicating that the drugs function properly. Phosphorylation of p70S6K1 was significantly induced at 48 and 72 h in cells treated with BMS-754807 or GSK1838705A (Fig. 1a, b). These findings suggest that the induction of p70S6K1 phosphorylation is through inhibition of IGF-1R. It has been reported that mutations in K-Ras and PIK3CA frequently occur in colorectal cancer, ~35–45% and 20%, respectively.^22,23^ Since IGF-1R governs PI3K/AKT and Ras/ERK signaling pathways,^1^ we next to test whether induction of p70S6K1 phosphorylation is dependent on K-Ras or PIK3CA mutation. Beside HCT116 cells (K-Ras mutant, PIK3CA mutant),^24^ we treated SW480 (K-Ras mutant, PIK3CA WT),^24^ LoVo (K-Ras mutant, PIK3CA WT),^24^ and RKO (K-Ras WT, PIK3CA mutant)^24^ cells with BMS-754807 (240 nM) for 0–72 h. The p70S6K1 phosphorylation was induced in all cells treated with BMS-754807 for 48–72 h (Fig. 1c, d), indicating that induction of p70S6K1 phosphorylation by inhibition of IGF-1R is independent of K-Ras or PIK3CA mutations.

### IGF-1R inhibition-induced p70S6K1 phosphorylation contributes to cell survival

**Fig. 2:**
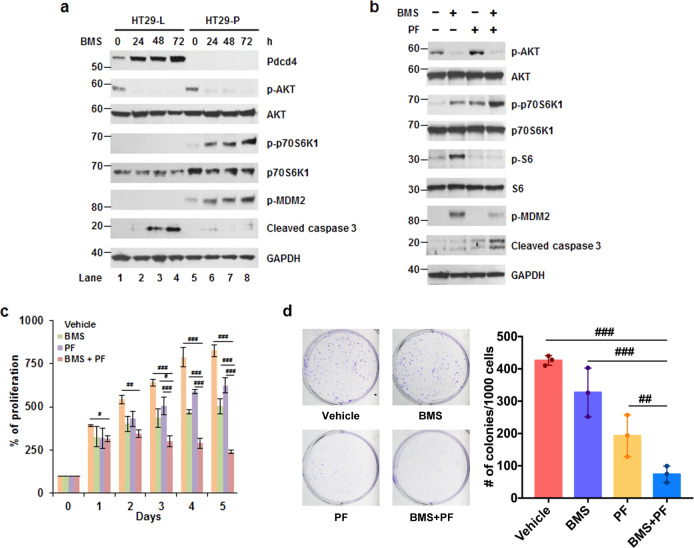
BMS-754807-induced p70S6K1 phosphorylation contributes to cell survival. **a** BMS-754807 induced p70S6K1 phosphorylation in Pdcd4 knockdown cells. Western blot analyses were performed using cell extracts from control (HT29-L) and Pdcd4 knockdown (HT29-P) cells treated with BMS-754807 (240 nM) for 0–72 h. Representative images are shown. **b** A combination of BMS-754807 and PF-4708671 reversed the BMS-754807-induced p70S6K1 activation and increased the cleaved caspase 3 level. Western blot analyses were performed using cell extracts from cells treated with vehicle, BMS-754807 (240 nM), PF-4708671(10 μM), and BMS-754807 (240 nM)+PF-4708671 (10 μM) for 72 h. Representative images are shown. **c**, **d** The combination of BMS-754807 and PF-4708671 inhibits the proliferation and colony formation. **c** HCT116 cells were treated with vehicle, BMS-754807 (240 nM), PF-4708671 (10 μM), BMS-754807 (240 nM)+PF-4708671 (10 μM) for 0–5 days. Cell viability was determined by XTT. The absorbance at day 0 is designated as 100%. Data from four replicates were analyzed one-way ANOVA with Dunnett’s multiple comparison (mean ± SD; ^#^*P* < 0.05; ^##^*P* < 0.01; ^###^*P* < 0.001). **d** Cells were treated with vehicle, BMS-754807 (240 nM), PF-4708671 (10 μM), or BMS-754807 (240 nM)+PF-708671 (10 μM) for 7 days. Data from three replicates were analyzed by one-way ANOVA with Dunnett’s multiple comparison (mean ± SD; ^##^*P* < 0.01; ^###^*P* < 0.001). BMS: BMS-574807; PF: 4708671

Previously, we showed that Pdcd4 suppresses IGF-1R inhibition-induced p70S6K1 phosphorylation when cells were treated with the IGF-1R inhibitor, OSI-906.^10^ To test whether downregulation of Pdcd4 enhanced p70S6K1 phosphorylation by BMS-754807 to promote cell survival, we treated Pdcd4 knockdown (HT29-P) and *LacZ* shRNA transfected control (HT29-L) cells with BMS-754807 for 0–72 h. As expected, Pdcd4 knockdown cells (HT29-P) dramatically induced p70S6K1 phosphorylation when cells were treated with BMS-754807 for 24–72 h (Fig. 2a and Supplementary Fig. 1). As a consequence, the phosphorylation of the p70S6K1 downstream target, MDM2, also elevated with BMS-754807 treatment (Fig. 2a and Supplementary Fig. 1). Consistent with the increase in survival signal, i.e., phosphorylation of p70S6K1 and MDM2, the apoptotic protein, cleaved caspase 3, significantly decreased in the HT29-P cells comparing to that in the HT29-L cells (Fig. 2a, lanes 3 and 4 vs lanes 7 and 8). To further confirm the inhibition of BMS-754807-induced p70S6K1 activation leading to cell death, we treated HCT116 cells with BMS-754807, PF-4708671 (p70S6K inhibitor), or both. As shown in Fig. 2b, cells treated with both BMS-754807 and PF-4708671 reversed the BMS-754807-induced phosphorylation of MDM2 and increased the levels of cleaved caspase 3. Besides, HCT116 cells treated with both BMS-754807 and PF-4708671 significantly decreased in proliferation (Fig. 2c) and the number of colonies (Fig. 2d) comparing with vehicle control, BMS-754807, or PF-4708671. These findings suggest that the induction of p70S6K1 phosphorylation is the key event for cell survival in BMS-754807 treatment. It is noteworthy that PF-4708671 can inhibit p70S6K1 as shown by the diminished phosphorylation of its downstream target, ribosomal protein S6, even though the level of p70S6K1 phosphorylation increased (Fig. 2b).^25^

### Knockdown of Pdcd4 increases p70S6K1 translation

**Fig. 3:**
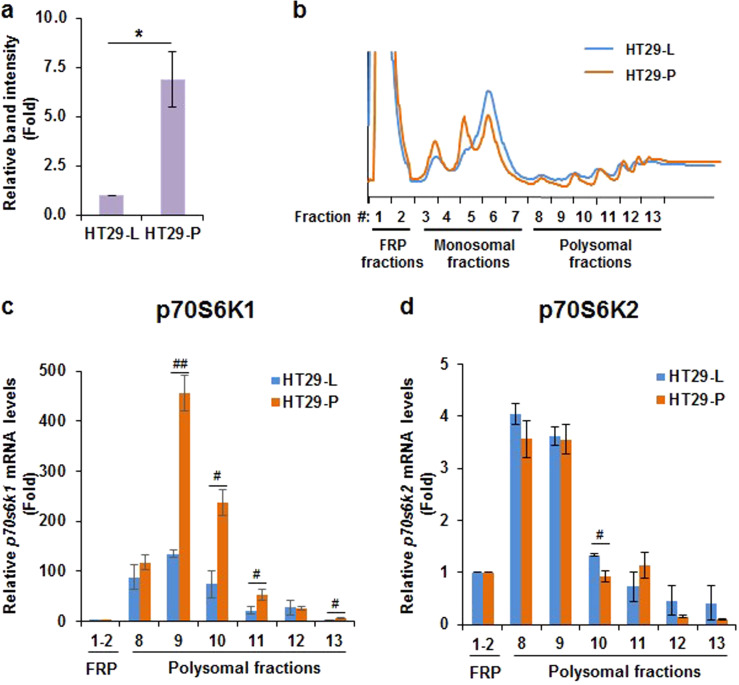
Knockdown of Pdcd4 enhances p70S6K1 translation. **a** p70S6K1 protein levels were increased in Pdcd4 knockdown cells. The level of p70S6K1 in control (HT29-L) and Pdcd4 knockdown (HT29-P) cells at 0 h treatment in Fig. 2a was quantified. The band intensity of p70S6K1/GAPDH in HT29-L cells is designated as 1. Data from three independent experiments were analyzed by one-sample *t-*test (mean ± SD; *P < 0.05). **b** Polysomal profiles of HT29-L and HT29-P cells. Pdcd4 knockdown increases *p70s6k1* mRNA (**c**) but not *p70s6k2* mRNA (**d**) in polysomal fractions. The cell lysates from HT29-L and HT29-P cells were subjected to sucrose gradient fractionation. After fractionation, the mRNAs in each fraction were purified and quantified with RT-qPCR. RT-qPCR was performed with three replicates to determine the relative levels of *p70S6K1* or *p70S6K2* mRNA by comparison of *p70S6K1* or *p70S6K2* mRNA in each polysomal fraction to that in corresponding pooled free RNAs and proteins (FRP) fraction, respectively. Data from two independent experiments were analyzed by two-sample *t-*test (mean ± SD*;* ^#^*P* < 0.05; ^##^*P* < 0.01)

Besides the enhanced phosphorylation of p70S6K1 by Pdcd4 knockdown, we noticed that the protein level of p70S6K1 also increased (Fig. 2a, lane 1 vs. 5 and Fig. 3a). Since Pdcd4 functions as a protein translation inhibitor,^13^ it is possible that Pdcd4 knockdown promotes p70S6K1 translation and thereby increases the p70S6K1 protein level. To test it, a sucrose gradient fractionation was performed to separate free RNAs, monosomes, and polysomes (Fig. 3b). During protein translation, the actively translated mRNAs associated with many ribosomes to form polysomes. Thus, mRNAs distributed in the polysomal fractions are the ones actively translated. After fractionation, the mRNAs in each polysomal fraction (fractions #8–13) were purified and quantified with RT-qPCR. As shown in Fig. 3c, knockdown of Pdcd4 increased the *p70S6K1* mRNA population in the polysomal fractions. Among these polysomal fractions, the level of *p70s6k1* mRNA significantly increased in the fraction #9, #10, #11, and #13 of HT29-P cells as compared to the corresponding fraction of HT29-L cells (Fig. 3c). By contrast, the levels of *p70s6k2* mRNAs in the polysomal fractions of HT29-P cells were similar to those of the HT29-L cells or 30% decrease in the fraction #10 (Fig. 3d). These results indicate that Pdcd4 knockdown promotes p70S6K1 translation.

### Inhibition of AKT induces MEK1/2 phosphorylation but not ERK1/2 phosphorylation

**Fig. 4:**
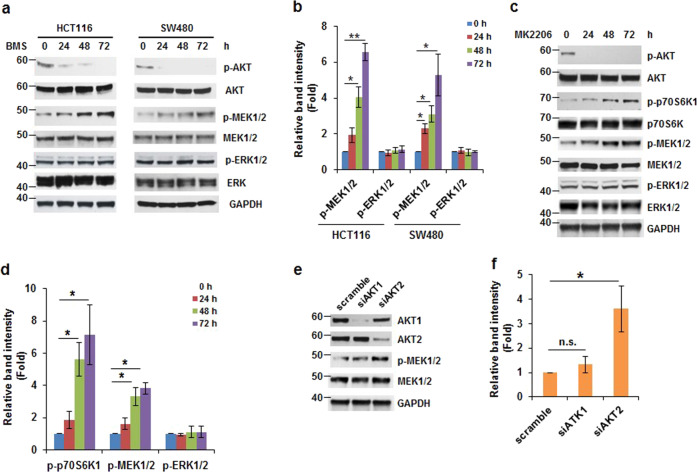
Inhibition of AKT induces phosphorylation of MEK1/2 but not ERK1/2. **a**, **b** BMS-754807 induces MEK1/2 phosphorylation in colon tumor cells. Western blot analyses of MEK1/2 and ERK1/2 phosphorylation using extracts from cells treated with BMS-754807 (240 nM) for 0–72 h. **a** Representative images are shown. **b** Densitometric quantification of the levels of phospho-MEK and phoshpo-ERK in (**a**). The ratio of phospho-MEK/GAPDH or phospho-ERK/GAPDH in HCT116 or SW480 cells with 0 h treatment is designated as 1. Data from three independent experiments were analyzed by one-sample *t*-test (mean ± SD; *P < 0.05; **P < 0.01). **c**, **d** The inhibition of AKT induces phosphorylation of MEK1/2. Western blot analyses using extracts from cells treated with MK-2206 (0.5 μM) for 0–72 h. **c** Representative images are shown. **d** Densitometric quantification of levels of phospho-p70S6K1, phospho-MEK1/2, and phoshpo-ERK1/2 in (**c**). The ratio of phospho-p70S6K1/GAPDH, phospho-MEK/GAPDH, or phospho-ERK/GAPDH at 0 h treatment is designated as 1. Data from three independent experiments were analyzed from by one-sample *t*-test (mean ± SD; *P < 0.05; **P < 0.01)**. e**, **f** AKT2 knockdown increases MEK1/2 phosphorylation. HCT116 cells were transfected with scrambled siRNA, *akt1* siRNA, or *akt2* siRNA. Forty-eight hours post transfection, cell lysates were assayed by western blot. **e** Representative western blot images are shown. **f** Densitometric quantification of phospho-MEK1/2 levels in (**e**). The ratio of phospho-MEK/GAPDH of transfection with scrambled siRNA is designated as 1. Data from three independent experiments were analyzed by one-sample *t*-test (mean ± SD; *P < 0.05; n.s. no significance)

IGF-1R inhibition has been shown to inhibit PI3K/AKT and Ras/ERK signaling pathways.^26^ However, previous studies showed that ERK phosphorylation is barely suppressed in several colon tumor cell lines treated with IGF-1R inhibitor, OSI-906.^27^ Thus, it is interesting to examine the effect of BMS-754807 on ERK phosphorylation. As shown in Fig. 4a, the BMS-754807 functioned properly because it efficiently inhibited AKT phosphorylation at Ser473 in both HCT116 and SW480 cells. However, 72-h treatment of BMS-754807 in HCT116 and SW480 cells did not change the levels of phosphorylation of ERK1/2, showing BMS-754807 not able to suppress ERK1/2 activation (Fig. 4a, b). Surprisingly, the phosphorylation of MEK1/2, the upstream regulator of ERK1/2, was induced in HCT116 and SW480 cells treated with BMS-754807 for 48–72 h (Fig. 4a, b). We also found that the phosphorylation of JNK and p38, two stress-induced kinases, were barely detectable in the HCT116 cells treated with BMS-754807 for 72 h (Supplementary Fig. 3), suggesting that IGF-1R inhibition did not alter JNK and p38 signaling pathways. Our results indicate that colon HCT116 and SW480 cells treated with BMS-754807 induces phosphorylation of MEK1/2 but not ERK1/2.

Since BMS-754807 efficiently inhibited AKT phosphorylation at Ser473 (Fig. 4a), we assumed that MEK1/2 phosphorylation might be induced by inhibition of AKT activation. To test it, we treated HCT116 cells with AKT inhibitor, MK-2206 (0.5 μM), for 0–72 h. As expected, the phosphorylation of AKT at Ser473 was barely detectable after MK-2206 treatment (Fig. 4c). As the BMS-754807 treatment, cells treated with MK-2206 for 48 and 72 h increased MEK1/2 phosphorylation ~3- to 4-fold but did not affect ERK phosphorylation (Fig. 4c, d). In addition, the phosphorylation of p70S6K1 significantly elevated by MK-2206 treatment for 48 and 72 h (Fig. 4c, d). To further confirm that AKT inactivation increased MEK1/2 phosphorylation, we knocked down AKT1 and AKT2 expression by transfection of *akt1* and *akt2* siRNAs. As shown in Fig. 4e, transfection of *akt1* and *akt2* siRNA into HCT116 cells significantly reduced the expression of AKT1 and AKT2, respectively. Interestingly, knockdown of AKT2 but not AKT1 increased ~3.5-fold of MEK1/2 phosphorylation compared with the scrambled siRNA transfected cells (Fig. 4e, f). Taken together, our findings (Fig. 4) suggested that inhibition of AKT2 increases MEK1/2 phosphorylation in colon tumor cells.

### Inhibition of MEK1/2 increases AKT phosphorylation

**Fig. 5:**
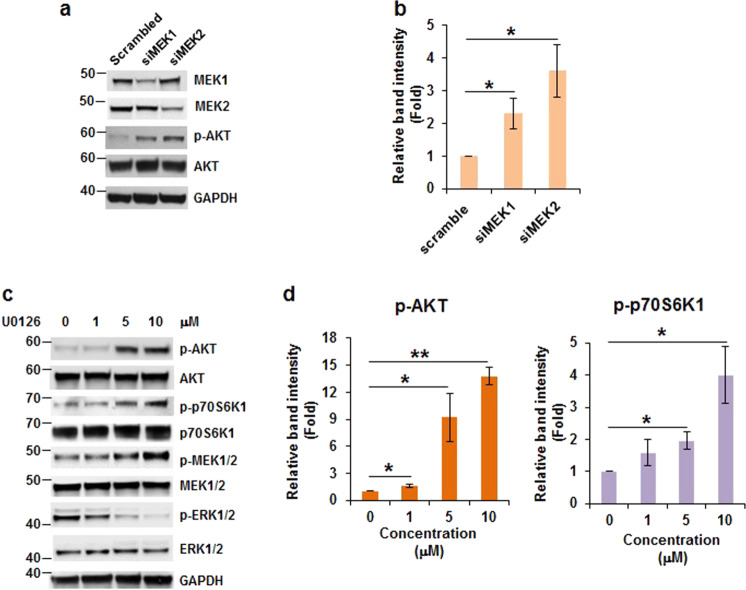
Inactivation of MEK1 or MEK2 stimulates AKT phosphorylation. **a**, **b** Knockdown of MEK1 or MEK2 boosts AKT phosphorylation. Cells were transfected with scrambled siRNA, *mek1* siRNA, or *mek2* siRNA. After 48 h, cell lysates were subjected to western blot analysis. **a** Representative images are shown. **b** Densitometric quantification of phospho-AKT levels in (**a**). The ratio of phospho-AKT/GAPDH of transfection with scrambled siRNA is designated as 1. Data from three independent experiments were analyzed by one-sample *t*-test (mean ± SD; *P < 0.05). **c**, **d** Inhibition of MEK1/2 activation induces AKT phosphorylation. HCT116 cells were treated with 0, 1, 5, and 10 μM of U0126 for 72 h. The cell lysates were used to examine the levels of phospho-AKT. **c** Representative western blot images are shown. **b** Densitometric quantification of phospho-AKT and phospho-p70S6K1 levels in (**c**). The ratio of phospho-AKT/GAPDH or phospho-p70S6K1 in cells with 0 μM of U0126 is designated as 1. Data from three independent experiments were analyzed by one-sample *t*-test (mean ± SD; *P < 0.05; **P < 0.01)

To test whether MEK inhibition can increase AKT phosphorylation to activate AKT, we knocked down MEK1 and MEK2 expressions with *mek1* and *mek2* siRNA, respectively. Phosphorylation of AKT at Ser473 was induced two- to fourfold by knockdown of MEK1 or MEK2 comparing to that in control cells (Fig. 5a, b). To verify the above observation, cells were treated with MEK inhibitor, U0126 (0–10 μM) and subsequently assayed for the status of AKT phosphorylation. AKT phosphorylation at Ser473 did not change in cells treated with 1 μM of U0126 but dramatically increased when cells were treated with 5 or 10 μM of U0126 (Fig. 5c, d). However, AKT phosphorylation at Thr308 was not detectable in HCT116 cells (data not shown). As expected, p70S6K1 phosphorylation also increased in cells treated with 5 and 10 μM of U0126 (Fig. 5c, d). These findings suggest that downregulation or inactivation of MEK activates AKT/p70S6K axis. Remarkably, U0126 increased the phosphorylation of MEK1/2 but inhibited ERK1/2 phosphorylation in HCT116 cells, suggesting that U0126 functioned properly. This phenomenon was also observed in H1299, A549, and MCF7 cells treated with 10 µM of U0126 for 0–72 h (Supplementary Fig. 4). Thus, it is possible that U0126 inhibited MEK kinase activity but increased its phosphorylation as seen in p70SK6 inhibitor, PF-4708671.^25^ Interestingly, the phosphorylation of ERK1/2 was inhibited by U0126 (Fig. 5c) but not IGF-1R inhibitors (Fig. 4a and ref. ^27^). This discrepancy may be due to the different mechanisms employed by different inhibitors to inhibit MEK1/2 activity, which requires further investigations.

### The combination of BMS-704807 and U0126 efficiently suppresses the growth and induces cell death in colon tumor cells

**Fig. 6:**
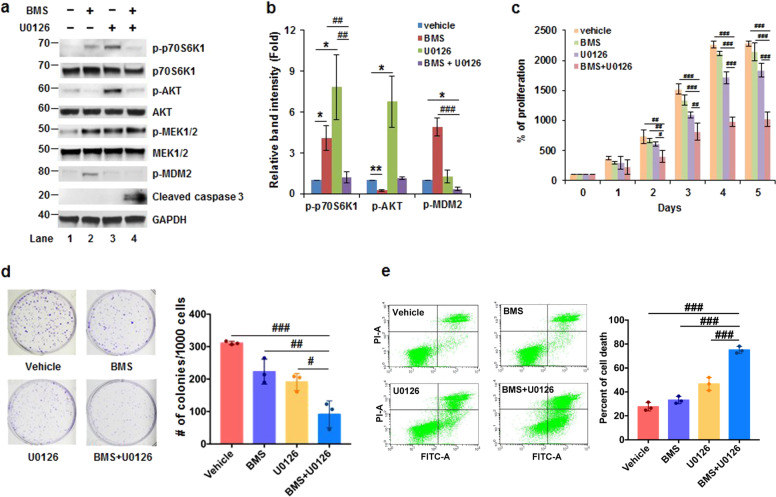
The combination of BMS-754807 and U0126 significantly inhibits the growth in colon tumor cells. **a**, **b** Inhibition of MEK1/2 suppresses BMS-754807-induced p70S6K1 phosphorylation and increases cleaved caspase 3. Cells were treated with vehicle (DMSO), BMS-754807, U0126, or BMS-754807 + U0126 for 72 h and subsequently used for western blot assays. **a** Representative images are shown. **b** Densitometric quantification of levels of phospho-p70S6K1, phospho-MDM2, and cleaved caspase 3 in (**a**). The ratio of phospho-p-70S6K1/GAPDH, phospho-MDM2/GAPDH or cleaved caspase 3/GAPDH in vehicle-treated cells is designated as 1. Data from three independent experiments were analyzed by one-sample *t*-test (*n* = 3; mean ± SD; *P < 0.05; **P < 0.01) or two-sample *t*-test (mean ± SD; ^##^P < 0.01; ^###^*P* < 0.001). **c** Proliferation assay. Cells were treated with indicated drugs for 0–5 days and the viability was measured with XTT. The absorbance at day 0 is designated as 100%. Data from four replicates were analyzed by one-way ANOVA with Dunnett’s multiple comparison (mean ± SD; ^#^*P* < 0.05; ^##^*P* < 0.01; ^###^*P* < 0.001). **d** Clonogenic assay. Cells were treated with indicated drugs for 7 days. Data from three replicates were analyzed by one-way ANOVA with Dunnett’s multiple comparison (mean ± SD; ^#^*P* < 0.05; ##*P* < 0.01; ^###^*P* < 0.001). The representative images are shown at the right. **e** Apoptosis assay. Cells were treated with the indicated drugs for 72 h, and the apoptotic cells were assessed using Annexin V assays. Data from three replicates were analyzed by one-way ANOVA with Dunnett’s multiple comparison (mean ± SD; ^###^*P* < 0.001). The representative images are shown at the right. BMS: BMS-754807

Our results suggested that activation of MEK1/2 is a key event for the induction of p70S6K1 phosphorylation in resistance to IGF-1R inhibition. We, next, examined whether inhibition of MEK1/2 leads to suppression of IGF-1R inhibition-induced p70S6K1 activation and promotes cell death. We treated HCT116 cells with vehicle (DMSO), BMS-754807 (240 nM), U0126 (5 μM), and BMS-754807 (240 nM)+U0126 (5 μM) for 3 days. As shown in Fig. 6a, b, HCT116 cells treated with BMS-754807 and U0126 induced p70S6K1 phosphorylation (lanes 2 and 3). The induction of p70S6K1 phosphorylation was abolished when cells were treated with both BMS-754807 and U0126 (Lane 4), indicating that suppression of MEK1/2 abolishes BMS-754807-induced p70S6K1 phosphorylation. As a consequence of p70S6K1 inhibition, the phosphorylation of MDM2 decreased and cleaved caspase 3 increased (Fig. 6a, b). To further validate whether a combination of BMS-754807 and U0126 efficiently inhibits proliferation and induces cell death, we performed cell growth and apoptosis assays. As shown in Fig. 6c, cells treated with BMS-754807 or U0126 slightly inhibited cell growth. In contrast, the cell growth was significantly inhibited with the combination of BMS-754807 and U0126 comparing with vehicle control, BMS-754807, or U0126 alone. Besides, the colony number also significantly reduced in cells treated with BMS-754807 + U0126 comparing to the vehicle, BMS-754807, or U0126 treated cells (Fig. 6d). Consistent with the growth inhibition, cell death significantly increased in BMS-754807 + U0126 treated cells than that in vehicle, BMS-754807, or U0126 treated cells (Fig. 6e).

**Fig. 7:**
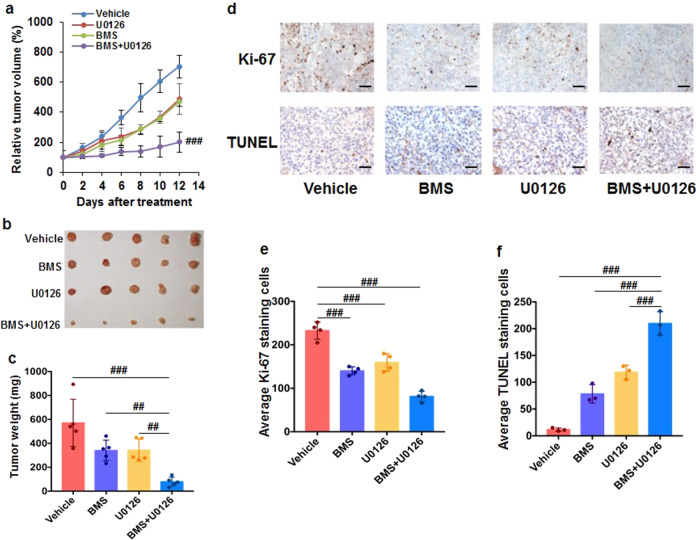
The combination of BMS-754807 and U0126 significantly suppresses tumor growth in the HCT116 colon xenograft model. NCr nude mice (five mice per group) were treated with vehicle [10% (v/v) DMSO in corn oil], BMS-754807 (25 mg/kg, BMS), U0126 (15 mg/kg), or BMS-754807 + U0126 (25 mg/kg+15 mg/kg) for 12 days. **a** The tumor volume was measured every other day with calipers. Data were analyzed by one-way ANOVA with Dunnett’s multiple comparison versus vehicle, BMS-754807, or U0126 after 12-day treatment (mean ± SD; ^###^*P* < 0.001). **b** Image of tumors after 12-day treatment. **c** The BMS-754807 + U0126 treatment significantly lowered the average tumor weight comparing to the vehicle, BMS-754807, or U0126 treatment. Data were analyzed by one-way ANOVA with Dunnett’s multiple comparison (mean ± SD; ^##^*P* < 0.01; ^###^*P* < 0.001). **d** Tumor sections were examined for proliferation (stained with Ki-67) and apoptosis (stained with TUNEL). The representative images are shown; scale bar, 10 μm. **e**, **f** The number of positive-staining cells in tumors in each treatment was calculated as the average number of four random images. The data were analyzed by one-way ANOVA with Dunnett’s multiple comparison (mean ± SD; ^###^*P* < 0.001). BMS: BMS-754807

To confirm the results from cultured cells, we tested the efficacy of the combination of BMS-754807 and U0126 on suppressing tumor growth in HCT116 cells derived tumor xenograft. The average tumor volume in mice treated with BMS-754807 + U0126 increased about twofold during the 12-day treatment while the tumor volume increased about sevenfold in the vehicle-treated mice (Fig. 7a). The average tumor volume in combination-treated mice was ~50% of that in mice treated with BMS-754807 or U0126 alone (Fig. 7a). In consistent with the tumor volume, the average tumor weight from the combination-treated mice was ~40% of that in the mice treated with BMS-754807 or U0126 alone (Fig. 7b, c). To verify that BMS-754807 + U0126 enhances apoptosis and attenuates proliferation in vivo, the tumor tissue sections were subjected to the analysis of proliferation and apoptosis by staining with Ki-67 and TUNEL, respectively. As shown in Fig. 7d, the tumor tissues from mice treated with BMS-754807 + U0126 showed the least Ki-67 and the most TUNEL comparing to the tumors from the mice treated with vehicle control, BMS-754807, or U0126. The Ki-67 positive-staining cells in tumors from mice treated with BMS-754807, U0126, and BMS-754807 + U0126 were ~60%, 63%, and 40% of those in tumors from vehicle-treated mice, respectively. Conversely, the TUNEL positive-staining cells in tumors from BMS-754807, U0126, and BMS-754807 + U0126 treated mice were ~7-fold, 10-fold, and 19-fold higher than those from vehicle-treated mice, respectively. These in vitro and in vivo findings (Figs. 6 and 7) suggest that inhibition of MEK1/2 suppresses IGF-1R-inhibition-induced p70S6K1 activation in colon tumor cells, providing a new strategy for colon cancer therapy.

## Discussion

**Fig. 8:**
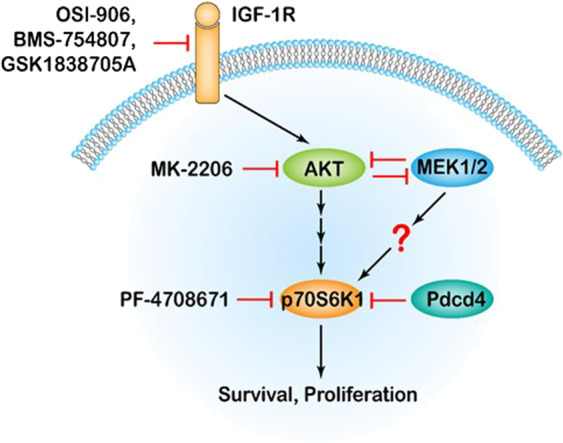
Schematic diagram showing that IGF-1R inhibition induces p70S6K1 activation to promote cell survival. Suppression of AKT activation by IGF-1R inhibitors (OSI-906, BMS-754807, and GSK1838705A) or AKT inhibitor (MK-2206) results in stimulation of MEK1/2 activity. Since AKT and MEK1/2 mutually repress each other, the inactivation of MEK1/2 activates AKT, which may lead to p70S6K1 phosphorylation to enhance cell survival. While activation of MEK1/2 represses AKT, but still increases p70S6K1 phosphorylation through an unknown mechanism. In addition, inhibition of p70S6K1 activation by PF-4708671 or Pdcd4 overcomes the BMS-754807-induced p70S6K1 activation and thus reduces cell survival

In this study, we found that extensive treatment of IGF-1R inhibitor induced phosphorylation of p70S6K1 in colon tumor cells (Fig. 1). Inhibition of p70S6K by PF-4708671 or suppression of p70S6K1 translation by Pdcd4 significantly increased the efficacy of BMS-754807 in promoting cell death and suppressing cell growth (Figs. 2 and 3). IGF-1R inhibitor elevated p70S6K1 phosphorylation, at least in part, through activation of MEK1/2 (Figs. 4 and 5), and the combination of the IGF-1R inhibitor and MEK inhibitor significantly suppressed cell growth and induced cell death in both cultured cells and mice (Figs. 6 and 7). Our findings provide a new mechanistic insight into the resistance of IGF-1R inhibition in which MEK1/2 activation stimulates the survival signal (Fig. 8).

p70S6K1 activation has been shown to enhance cell survival and reduce apoptosis.^28^ For example, p70S6K1 phosphorylates MDM2 at Ser166 resulting in polyubiquitination and degradation of p53 and thereby inhibits p53-dependent apoptosis.^12^ Inhibition of p70S6K1 results in the dephosphorylation of the pro-apoptotic protein, Bad, leading to mitochondria-dependent apoptosis.^29^ The finding that prolonged treatment with IGF-1R inhibitor induced p70S6K1 phosphorylation suggests the importance of p70S6K1 phosphorylation in cell survival when IGF-1R is inhibited (Fig. 1). In agreement with this scenario, inhibition of p70S6K1 activity by p70S6K inhibitor, PF4708971, significantly increased the cell sensitivity to IGF-1R inhibition via reducing the cell growth and colony formation (Fig. 2). Besides the colon cancer cells, the elevation of p70S6K1 phosphorylation is also observed in breast cancer MCF7 cells treated with an IGF-1R inhibitor, OSI-906.^30^ Collectively, our findings indicate that enhancement of p70S6K1 phosphorylation is a key event for the resistance to IGF-1R inhibition in colon cancer cells.

The result that Pdcd4 knockdown increased the distribution of *p70s6k1* mRNA but not *p70S6K2* mRNA in the polysomal fractions indicates Pdcd4 inhibiting p70S6K1 translation (Fig. 3). By binding with eIF4A, Pdcd4 inhibits eIF4A RNA helicase activity and thereby suppresses translation.^16^ During translation initiation, eIF4A’s helicase activity is essential to unwind the structured mRNA at 5′untranslated region (5′UTR), which allows the translation initiation complex moving from 5′ to 3′ direction to locate at the AUG codon.^17^ Thus, inhibition of eIF4A helicase activity results in suppression of translation, especially the translation of mRNAs with stable secondary structures at 5′UTR. Pdcd4 was initially demonstrated to preferentially inhibit translation of the luciferase mRNA possessing an artificial stem-loop structure at 5′UTR with the free energy of −44.8 kcal/mol.^31^ This concept was recently confirmed by the identification of Sin1 as a Pdcd4 target, whose mRNA forms a secondary structure at 5′UTR with the free energy of −145 kcal/mol.^19^ In addition to inhibiting eIF4A activity, Pdcd4 may repress protein translation through binding with RNA by preventing the formation of the translation initiation complex.^32^ Since the partial 5′UTR of *p70S6K1* mRNA forms a secondary structure with the free energy of −82.30 kcal/mol, it is most likely that Pdcd4 inhibits the p70S6K1 translation via repressing eIF4A activity. Yet, the mechanism needs to be further investigated. Interestingly, it has been reported that Pdcd4 is phosphorylated at Ser67 by p70S6K and followed by proteasomal degradation.^33^ Here, the result of Pdcd4 regulating p70S6K1 translation indicates a feedback regulation between Pdcd4 and p70S6K1.

Our data showed that inhibition of MEK1/2 by a pharmacological inhibitor (U0126) abolished the IGF-1R inhibition-induced p70S6K1 phosphorylation (Fig. 6). Inhibition of MEK1/2 by U0126 or MEK1/2 knockdown results in increased phosphorylation of AKT and p70S6K1 (Fig. 5). These findings indicate that the MEK1/2 activation is an essential event for p70S6K1 activation by IGF-1R inhibition. Activation of IGF-1R leads to the stimulation of two major downstream pathways, PI3K/AKT and Ras/ERK pathways.^1^ Previous studies have shown the crosstalk between Ras/ERK and PI3K/AKT pathway and thus inhibition of one pathway may induce activation of the other one.^34^ For example, in response to various stimulations, AKT negatively regulates ERK activation through inhibition of c-Raf phosphorylation.^35,36^ Conversely, inhibition of AKT activity by MN-2206 upregulates EKR phosphorylation in multiple myeloma cells.^37^ In agreement with this scenario, we observed that inhibition of AKT by BMS-754807 or MN-2206 notably increased the phosphorylation of MEK1/2 (Fig. 4a–d). Moreover, knockdown of AKT2 but not AKT1 significantly increased MEK1/2 phosphorylation (Fig. 4e, f). These findings indicate that the inactivation of AKT2 stimulates MEK1/2 activity in colon cancer cells resistant to IGF-1R inhibition. Also, inactivation of MEK1/2 by U0126 or knockdown of MEK1 or MEK2 increased AKT phosphorylation (Fig. 5), suggesting that AKT and MEK mutually repress each other. How does MEK activate AKT? Previous studies have demonstrated that MEK inhibition stimulates AKT activity through an ERK-mediated feedback loop activation.^37–39^ Since colon cancer cells treated with IGF-1R inhibitors do not change the ERK phosphorylation (Fig. 6a and ref. ^27^), ERK does not likely play a role in AKT activation. A recent study by Procaccia et al. suggested that AKT and MEK may directly bind each other.^40^ It is necessary to further investigate whether AKT and MEK bind each other to mutually suppress the counterpart.

Taken together, our results revealed that inhibition of AKT leads to activation of MEK1/2 to elevate p70S6K1 phosphorylation, while suppression of MEK1/2 results in the enhancement of AKT phosphorylation and thereby stimulation of p70S6K1 activity. Thus, phosphorylation of MEK1/2 is critical for p70S6K1 activation in colon cancer cells when IGF-1R is inhibited. Furthermore, a combination of IGF-1R inhibitor (BMS-754807) and MEK inhibitor (U0126) significantly suppressed cell viability and colony formation in cultured cells (Fig. 6) and tumor growth in mice (Fig. 7). Therefore, our data also provide a promising strategy of combining the IGF-1R and MEK1/2 inhibitors to suppress colon tumor growth. It is noteworthy that our data suggested inhibition of both IGF-1R and MEK1/2 attenuated p70S6K1 activation to promote cell death. Considering the complexity of crosstalk between signaling pathways, an unknown p70S6K independent mechanism might exist to promote cell survival, which is inhibited by the combination of IGF-1R and MEK1/2 inhibitors. This unknown mechanism needs further investigation.

## Materials and methods

### Reagents

BMS-754807, OSI-906, and GSK1838705A were obtained from Chemie Tek. MK-2206 and PF-4708671 were purchased from Selleckchem. U0126 was purchased from MedChemExpress. All drugs were dissolved in DMSO at a proper concentration and stored at −20 °C.

### Cell lines and culture

The HT29, HCT116, SW480, and LoVo cells were purchased from the ATCC and the RKO cell was a gift from Dr. Douglas Boyd (MD Anderson Cancer Center, Houston, TX). HT29, HCT116, and RKO cells were grown in McCoy’s medium (Invitrogen). LoVo and SW480 cells were cultured in RPMI-1640 medium (Hyclone). HT29-shLacZ (HT29-L) and HT29-shPdcd4 (HT29-P) cells were previously generated.^15^ Cells were cultured in the medium with 10% FBS, 2 mM l-glutamine, and 100 U/mL penicillin–streptomycin and incubated at 37 °C in a humidified atmosphere of 5% CO_2_ in air.

### Cell proliferation and clonogenic assays

The TACS XTT cell proliferation assay kit (Trevigen) was used for cell proliferation assays as described previously.^41^ Briefly, 5 × 10^3^ cells/well were seeded onto a 96-well plate 1 day before drug treatment. The sodium 3′-[1-(phenylaminocarbonyl)−3,4-tetrazolium]-bis (4-methoxy-6-nitro) benzene sulfonic acid hydrate (XTT) labeling mixture was added to the cell culture for 30 min and subsequently, the absorbance at 490 nm was measured using the iMark microplate reader (Bio-Rad Laboratories). For clonogenic assays, 1000 cells were seeded on a six-well plate and subsequently treated with drugs for 7 days. The number of colonies was determined after staining with 1% (w/v) crystal violet.

### Apoptosis assays

The Annexin V–FITC Apoptosis Kit (Biolegend) was used for apoptosis assays according to the manufacturer’s protocol. The percentage of apoptotic cells was measured by a FacsCalibur cell analyzer (BD Biosciences).

### Western blot analysis

Cells were lysed in lysis buffer [50 mM Tris-HCl (pH 7.5), 1% NP-40, 0.5% (v/v) sodium deoxycholate, 150 mM NaCl and 1× Halt protease and phosphatase inhibitor mixture (Pierce)] on ice for 1 h. Twenty to forty microgram of protein was separated onto 4–12% SurePAGE gel (Genscript) and transferred to nitrocellulose membrane. After incubation with primary antibodies followed by horseradish peroxidase-linked secondary antibody, the target protein was visualized by Immobilon Western Chemiluminescent HRP substrates (Millipore). The antibodies against phospho-AKT(Ser473) (4060), AKT(pan) (4691), phospho-p70S6K(Thr389) (9205), p70S6K (2708), phospho-ERK1/2(Thr202/Tyr204) (4370), ERK1/2 (4695), phospho-S6 ribosomal protein (Ser235/236) (4858), S6 ribosomal protein (2217), phospho-MDM2(Ser166) (3521), phospho-MEK1/2(Ser217/221) (9154), MEK1/2 (8727), MEK1 (12671), and cleaved caspase 3 (9664) were purchased from Cell Signaling (1:1000 or 1:2000 dilution). The MEK2 (67410–1-Ig) antibody was purchased from Proteintech (1:4,000 dilution). The GAPDH (sc-47724) antibody was purchased from Santa Cruz Biotechnology (1:5000 dilution).

### Sucrose gradient fractionation and polysome analysis

Sucrose gradient fractionation was carried out as described previously.^19^ Briefly, cycloheximide (10 μg/ml) was added into the culture medium and incubated for 10 min before cells were harvested. The cells were lysed in lysis buffer [10 mM Tris (pH 7.4), 10 mM KCl, 2 mM MgCl, 1 mM EGTA, 1 mM DTT, 0.05% (v/v) Triton X-100 and 1× protease inhibitor (Pierce)] for 30 min on ice. After centrifugation, the protein concentration of the supernatant was determined. An equal amount of protein in the supernatant was layered onto 10–45% (w/w) sucrose gradient and centrifuged at 36000 r.p.m. for 2 h to separated free RNAs and ribosome-bound RNAs. Fractions were collected using Piston Gradient Fractionator (Biocomp) and Econo UV monitor (Bio-Rad). Data were acquired by Gradient Profiler software (Biocomp).

### RNA extraction and RT-qPCR

Total RNA was extracted from each sucrose gradient fraction using Trizol reagent (Invitrogen). The first strand of cDNA was synthesized from each fraction using the Superscript First-Strand Synthesis System (ThermoFisher). The p70S6K1, p70S6K2, and GAPDH mRNA levels were quantified by qPCR using primers purchased from Qiagen. The relative *p70S6K1* or *p70S6K2* mRNA levels were calculated by the 2^−ΔΔCt^ method.^42^ The average Ct values of three replicates in each fraction for *p70S6K1* or *p70S6K2* mRNA was normalized to the average Ct value of internal control *GAPDH* mRNA using the formula ΔCt = Ct(p70S6K1)-Ct(GAPDH). The relative level of *p70S6K1* or *p70S6K2* mRNA was determined by comparison of *p70S6K1* or *p70S6K2* mRNA in each polysomal fraction to that in the corresponding free RNAs fraction, respectively, using the formula 2^−[ΔCt(polysomal fraction)−ΔCt(free RNAs fraction)]^.

### Knockdown of AKT and MEK

Cells (3.5 × 10^5^) were transfected with 110 pmol of *akt1*, *akt2, mek1*, or *mek2* DsiRNA (Integrated DNA Technologies) using INTERFERin *in vitro* siRNA transfection reagent (Polyplus) as instructed by the manufacturer’s protocol. After 48 h, cells were harvested for proliferation and Western blot analyses.

### In vivo xenograft study

Male and female athymic nude mice (CrTac:NCr-Foxn1nu) were purchased from Taconic at age of 4- to 5-week. The mice were housed under pathogen-free conditions with commercial diet, water ad libitum, and 12 h light/12 h dark cycle. The experimental protocol was approved by IACUC of the University of Kentucky based on the NIH guidelines. Two million HCT116 cells were injected s.c. into the flank of each mouse and the tumor size was monitored and determined as previously described.^10^ When tumors reached 50–150 mm^3^, mice were randomly divided into four groups (five mice per group) and i.p. injected with (i) vehicle [10% (v/v) DMSO in corn oil); (ii) BMS-754807 (25 mg/kg); (iii) U0126 (15 mg/kg); and (iv) BMS-754807 (25 mg/kg) + U0126 (15 mg/kg) every day for 12 days. All mice under these treatments showed no sign of toxicity, i.e., bodyweight loss more than 15%, diarrhea, or decreased food intake.

### Statistical analysis

Statistical analyses were performed using the one-sample *t*-test for quantification of the band intensity of western blots, the two-sample *t*-test for comparing the means of two groups, and the one-way ANOVA with Dunnett’s multiple comparison for comparing means among multiple groups. Data are shown as the mean ± standard deviation (SD). The difference is considered statistically significant at the *P* < 0.05 level.

## Supplementary information

Supplemental Materials
