# Supplementary material for: IGF-1R inhibition induces MEK phosphorylation to promote survival in colon carcinomas
Source: Signal Transduct Target Ther. 2020 Aug 26;5:153. doi: 10.1038/s41392-020-0204-0 (PMC7447751; doi:10.1038/s41392-020-0204-0)
Supplement: Supplementary file 1 — Supplemental Materials [file 41392_2020_204_MOESM1_ESM.pdf]

Supplementary Materials for

IGF-1R inhibition induces MEK phosphorylation to promote survival in colon carcinomas

Qing Wang, Yan Zhang, Jiang Zhu, Honggang Zheng, Shuntai Chen, Li Chen, and Hsin-Sheng Yang

Correspondence to:hyang3@uky.edu

This PDF file includes:

Figures. S1 to S4

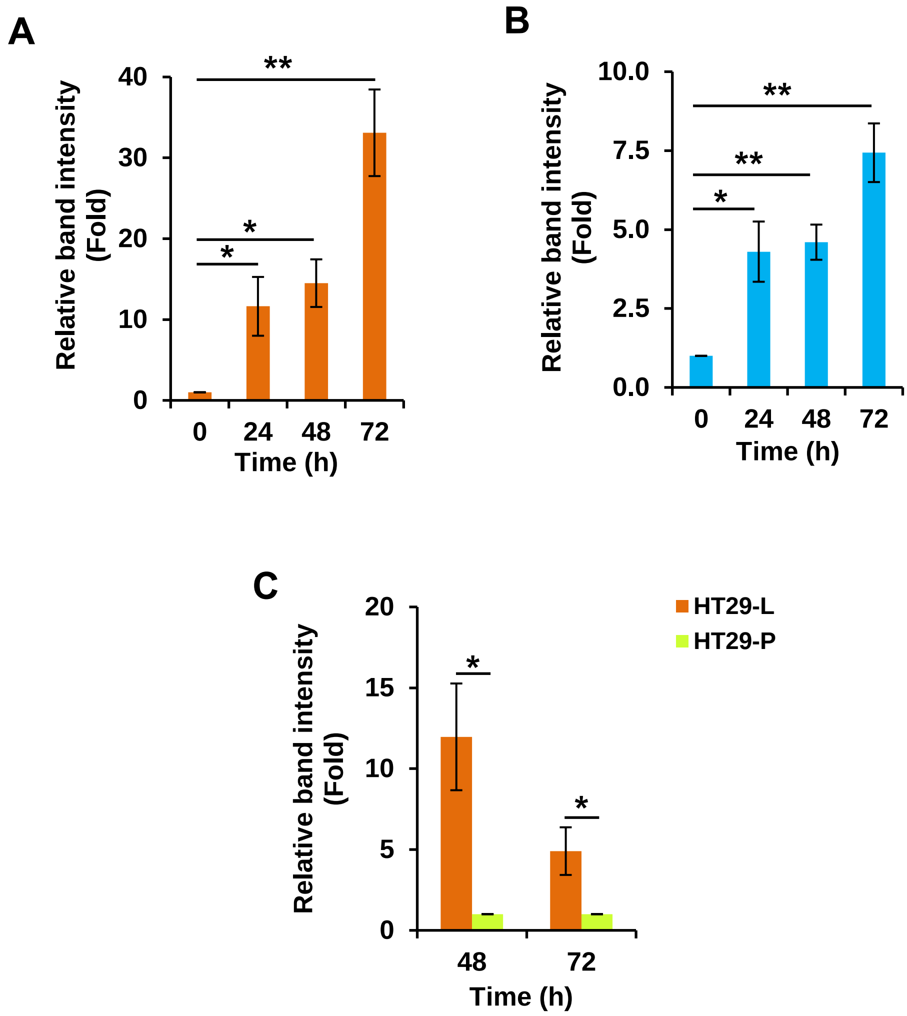

Figure S1. Densitometric quantification of the Western blot results in Fig. 2A. (A and B) The levels of phospho-p70S6K1 (A) and phospho-MDM-2 (B) in BMS-754807-treated HT29-P cells were quantified. The band intensity of 0 h treatment is designated as 1. (C) The levels of cleaved caspase 3 in BMS-754807 treated HT29-L and HT29-P cells were quantified. The band intensity of 48 and 72 h treatment in HT29-P cells is designated as 1. Data from three independent experiments were analyzed by one-sample *t*-test (mean ±SD; *, $P<0.05$; **, $P<0.01$).

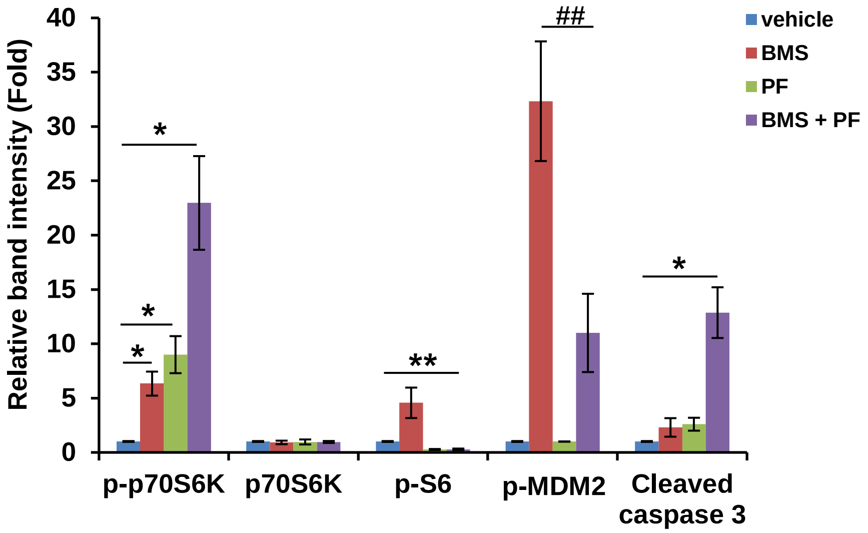

Figure. S2. Densitometric quantification of the Western blot results of phospho-p70S6K1, p70S6K1, phospho-S6, phospho-MDM2, and cleaved caspase 3 in Fig. 2B. The band intensity of 0 h treatment is designated as 1. Data from three independent experiments were analyzed by one-sample *t*-test (mean ± SD; *, P<0.05; **, P<0.01) or two-sample student's *t*-test (mean ± SD; ##, P<0.01).

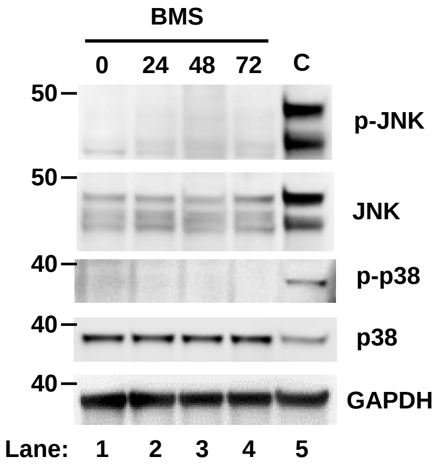

Figure. S3. BMS-754807 does not induce phosphorylation of JNK and p38 in HCT116 cells. HCT116 cells were treated with BMS-754807 (240 nM) for 0, 24, 48, 72 h (lane 1 to 4). The A549 cells treated with Platycodin D (10 µM) for 24 h to induce phosphorylation of JNK and p38 as a positive control (C, lane 5).[1] The antibodies against to phospho-JNK (4668), JNK (9252), phospho-p38 (9215) and p38 (9212) were purchased from Cell signaling.

1. Zhao, R., Chen, R, Jiang, Z., Zhao, F., Xi et al. Platycodin-D Induced Autophagy in Non-Small Cell Lung Cancer Cells via PI3K/Akt/mTOR and MAPK Signaling Pathways. *J. Cancer* **6**, 623–631 (2015).

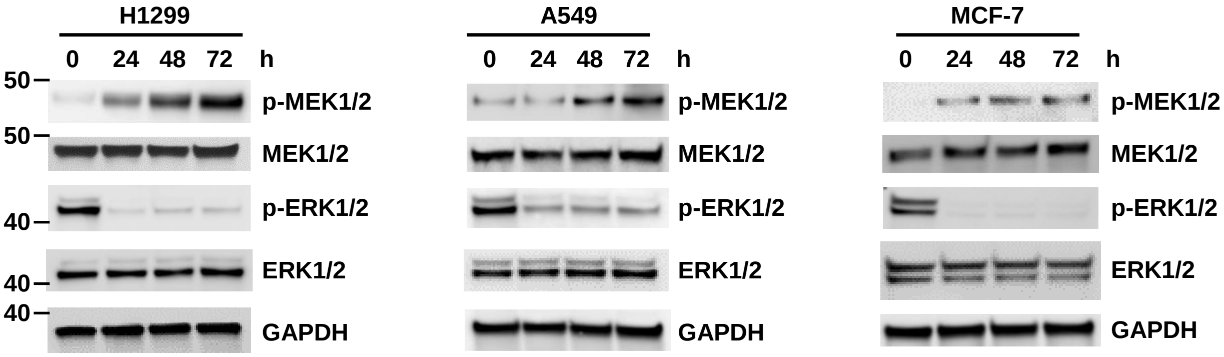

Figure. S4. U0126 increased MEK phosphorylation in H1299, A549, and MCF-7 cells. Cells were treated with U0126 (5 μM) for 0, 24, 48, 72 h. The antibodies against to phospho-MEK1/2 (9154), MEK1/2 (8727), phospho-ERK1/2 (4370) and ERK1/2 (4695) were purchased from Cell signaling.
